# Supplemental Cellular Protection by a Carotenoid Extends Lifespan via Ins/IGF-1 Signaling in *Caenorhabditis elegans*

**DOI:** 10.1155/2011/596240

**Published:** 2011-10-12

**Authors:** Koumei Yazaki, Chinatsu Yoshikoshi, Satoru Oshiro, Sumino Yanase

**Affiliations:** Department of Health Science, Daito Bunka University School of Sports and Health Science, Iwadono 560, Higashi-matsuyama, Saitama 355-8501, Japan

## Abstract

Astaxanthin (AX), which is produced by some marine animals, is a type of carotenoid that has antioxidative properties. In this study, we initially examined the effects of AX on the aging of a model organism *C. elegans* that has the conserved intracellular pathways related to mammalian longevity. The continuous treatments with AX (0.1 to 1 mM) from both the prereproductive and young adult stages extended the mean lifespans by about 16–30% in the wild-type and long-lived mutant *age-1* of *C. elegans*. In contrast, the AX-dependent lifespan extension was not observed even in a *daf-16* null mutant. Especially, the expression of genes encoding superoxide dismutases and catalases increased in two weeks after hatching, and the DAF-16 protein was translocated to the nucleus in the AX-exposed wild type. These results suggest that AX protects the cell organelle mitochondria and nucleus of the nematode, resulting in a lifespan extension via an Ins/IGF-1 signaling pathway during normal aging, at least in part.

## 1. Introduction

It has been understood that antioxidants and free radical scavengers decrease the intracellular reactive oxygen species (ROS) in treated experimental organisms and prolong their lifespans based on the free radical theory of aging [1, 2]. In the model organism nematode, *Caenorhabditis elegans* (*C. elegans*), there are many reports that dietary supplements, such as antioxidants and radical scavengers, extended the lifespan. *C. elegans* is an excellent experimental system to assess the pharmacological influence on intracellular aging pathways conserved between invertebrates and vertebrates [3, 4]. For example, it is conceivable that antioxidants, such as vitamin E, simply act to reduce the intracellular ROS in *C. elegans* [5]. The flavonoids, such as quercetin, are made to decrease the accumulation of the aging marker lipofuscin and localize the DAF-16 transcription factor, a homolog of mammalian FoxO, in the nucleus from the cytosol via an insulin/insulin-like growth factor-1 (Ins/IGF-1) signaling [6]. Oxaloacetate, the citric acid cycle metabolite, also increased the lifespan through an AMPK/FOXO-dependent pathway [7]. CoQ_10_, which is essential for the mitochondrial respiratory chain, reduced the superoxide anion levels mainly produced during electron transport [8]. In contrast, resveratrol that is a polyphenol found in red wine mimics calorie restriction by stimulating sirtuins, increasing the DNA stability, and extending the lifespan of metazoans [9–11]. Thus, molecular mechanisms of the supplemental lifespan extension are classified based on several intracellular pathways evolutionarily conserved from yeast to mammals.

 Environmental effects on the nematode, which changes the DNA structure and repair, behavior, genetic recombination frequency, oxygen (O_2_) consumption, and lipofuscin accumulation over its lifespan, are very important when considering the lifespan extension [12]. It is estimated that the heritability of the lifespan in *C. elegans* is between 20% and 50%, and the remaining percentage is mainly due to environmental effects, including nutrients and pathogens in the medium and ROS resources such as O_2_ and ionizing radiation (IR) in atmosphere, on the lifespan [13]. Therefore, the environmental effects on the lifespan of worms are not negligible. During its growth, the worm continues to intake nutrients from the medium with or without the bacterium *Escherichia coli* (*E. coli*) as the food source. The length of the mean lifespan as a worm group is remarkably affected by the environmental nutrients in the culturing medium because the *C. elegans* genome has a nearly uniform base composition [14]. These environmental effects can be classified into factors that shorten or extend the nematode lifespan upon their exposure or lack of exposure. 

 On the other hand, the enzymatic antioxidant systems in *C. elegans*, for example, superoxide dismutase (SOD) and catalase, play an important role in protecting living cells from ROS. SOD and catalase scavenge the intracellular superoxide radical (^∙^O_2_^−^) and hydrogen peroxide (H_2_O_2_), respectively. In *C. elegans*, five genes encoding these SODs (*sod-1* to *sod-5*) and three genes encoding these catalases (*ctl-1* to *ctl-3*) have been identified in the genome [15–21]. *sod-1* and *sod-5* genes encode cytosolic Cu/Zn SODs, *sod-2* and *sod-3* genes encode mitochondrial Mn SODs, and *sod-4* gene encodes the homolog of the extracellular Cu/Zn SOD in mammals. *ctl-1* and *ctl-3* genes encode unusual cytosolic catalases, and *ctl-2* gene encodes a peroxisomal catalase. In these genes, *sod-3*, *sod-5*, *ctl-1,* and *ctl-2* are direct targets of the transcription factor DAF-16, which is a key regulator of the Ins/IGF-1 signaling pathway implicated in the normal aging process of *C. elegans *[22–24]. Expressions of these subsets of antioxidant genes in *C. elegans* are also induced by the exposure to dietary supplements via the Ins/IGF-1 signaling pathway [6]. 

 Astaxanthin (AX), which is produced by marine animals, is a kind of carotenoid and shows a strong antioxidant activity that is attributed to the quenching of singlet oxygen (^1^O_2_) and the scavenging of lipid peroxidation by free radicals. In addition, AX inhibits the production of lipid peroxides in the animal cell membranes, and the antioxidant activity is about 2-fold more effective than *β*-carotene. The efficient antioxidant activity of AX is suggested to be due to the unique conjugated polyene structure of the terminal ring moiety [25]. It is reported that a marine carotenoid, fucoxanthin (FX), improved the insulin resistance and decreased the blood glucose level in mammals through the downregulation of the tumor necrosis factor-*α* [26]. Thus, the specific regulation of carotenoids containing AX and FX on the intracellular biomolecules is responsible for the characteristic chemical structures, which differ depending on the length of the polyene chain, a long conjugated double bond system forming the backbone of the molecule. We report the effects of AX, which has not only a strong antioxidant activity but also some biological activities that affect the nematode *C. elegans *lifespan.

## 2. Results

**Figure 1:**
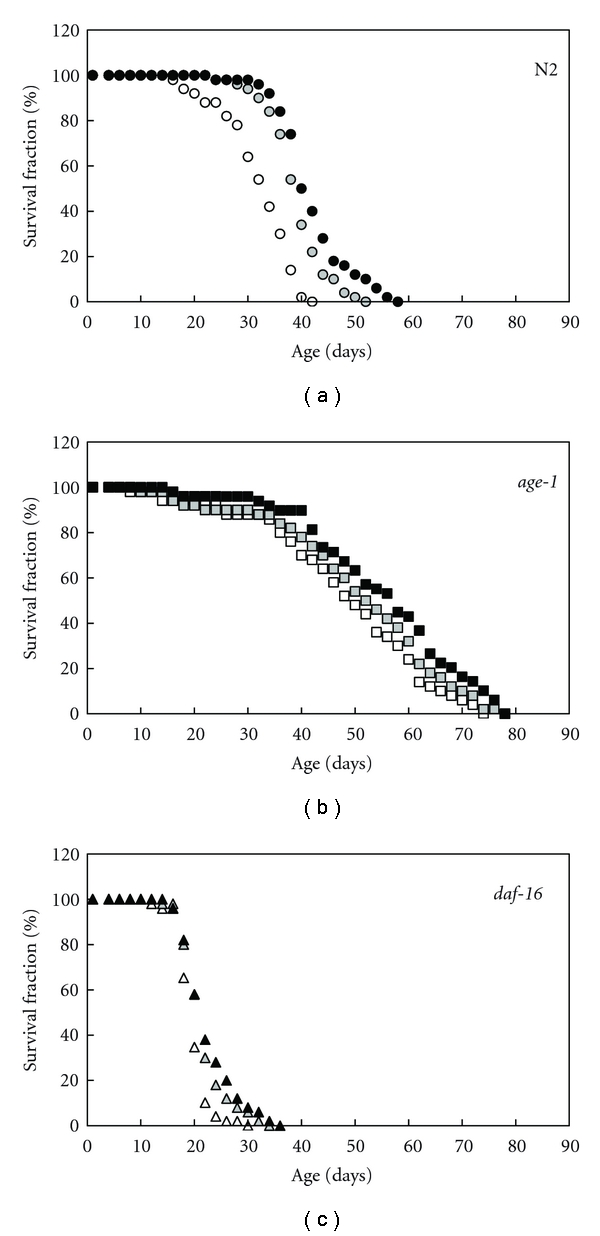
Survival curves at 20°C in wild-type N2, *age-1(hx546)*, and *daf-16(mgDf50)* animals. About 100 animals were used in each experiment. Open circle, square, and triangle show controls, shaded circle, square, and triangle show the treatment with 0.1 mM AX, and closed circle, square, and triangle show the treatment with 1 mM AX. Means of lifespan ± standard deviation (SD) in control, 0.1 mM, and 1 mM AX were as Table 1.

Continuous treatment with 0.1 to 1 mM AX from each stage of the first-stage larva (L1) or young adult in the hermaphrodite extended about 16–30% each mean of lifespan in the wild-type N2 and long-lived mutant *age-1* of *C. elegans *(Figure 1 and Table 1). The AX-dependent lifespan extensions in N2 were more notable than these of the *age-1 * animals. Moreover, the maximum lifespan in N2 also increased significantly depending on the concentration of AX. In contrast, the AX-dependent increases in the mean and maximum lifespan were not statistically clear in a null allele of the *daf-16* gene mutant, *daf-16(mgDf50)* animals. The wild-type lifespans measured using AX, which had not been solubilized in DMSO, were not significantly extended in a preliminary experiment (data not shown). 

**Figure 2:**
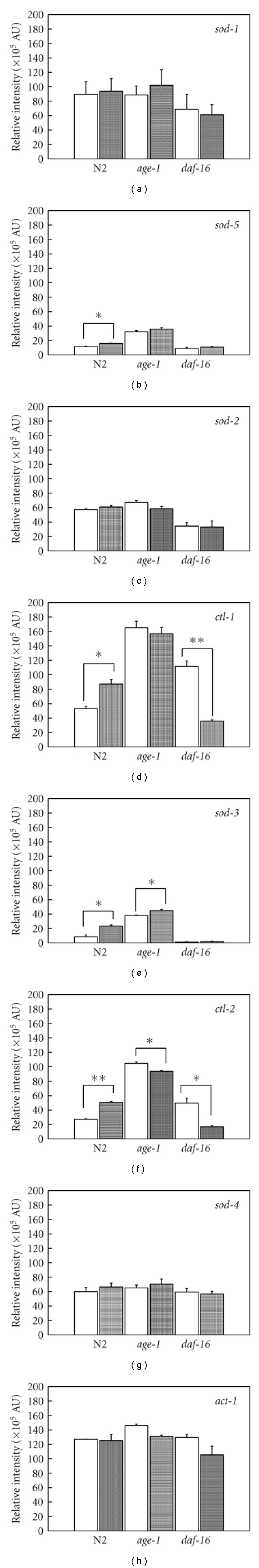
mRNA expression levels of *sod* and *ctl* genes in wild-type N2, *age-1(hx546)*, and *daf-16(mgDf50)* animals using RT-PCR. Each mRNA of 14-day-old animals was prepared and analyzed. Panels indicate the quantitative data obtained using the luminescent image analyzer and AU in the panels indicates arbitrary unit. Data are means ± SD of three or more independent experiments. Left-hand open column and right-hand shaded column for each strain indicate values without and with 1 mM AX-exposure, respectively. Asterisk indicates significant difference during the values without and with AX exposure. *P* values, which were calculated using a two-tailed *t*-test for paired samples with unequal variances, are *P < 0.05 and **P < 0.005.

On the other hand, the AX-dependent increases in the expression of some genes encoding antioxidant enzymes, such as SOD and catalase, were significantly observed in the wild-type N2 (Figure 2). Especially, the expression of the *sod-3*, *sod-5*, *ctl-1,* and *ctl-2* genes in the AX-treated N2 was significantly increased within two weeks after the AX treatment. All these genes are targets of the DAF-16 transcription factor, and the expression is regulated via the Ins/IGF-1 signaling pathway associated with oxidative stress resistance and aging in *C. elegans *[22–24]. The AX-dependent increases in the expression levels of these genes were not yet observed in the 7-day-old animals (data not shown). Thus, it was recognized that there are the time lags until the AX-dependent rising in the expression of these genes. In these genes; however, there are the genes that were regulated by other transcription factors (e.g., role of SKN-1 in *ctl*-genes) [27]. Therefore, the inconsistency during the intrinsic catalase activities in this paper and the mRNA levels in the previous data may depend on the diversity in transcriptional regulation of *ctl* genes expression [28]. 

**Figure 3:**
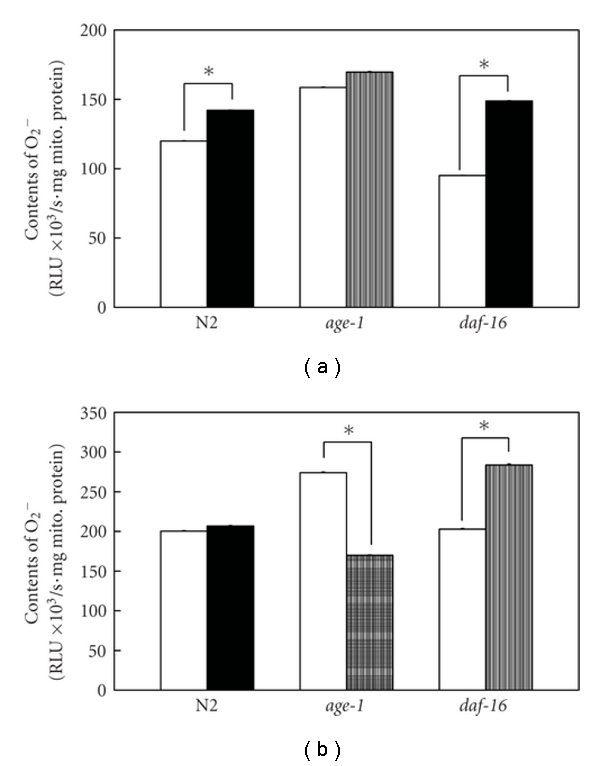
Mitochondrial ^∙^O_2_^−^ contents of various strains. Left-hand open bar for each strain of 4 days old (a) and 14 days old (b) indicates mitochondrial ^∙^O_2_^−^ level *in vitro* without 1 mM AX exposure, and right-hand shaded bar indicates values with AX exposure. Data are means ± standard error of the mean (SEM) of ten independent measurements. *P* values, which were calculated using a two-tailed *t*-test for paired samples with unequal variances, are *P < 0.001.

Furthermore, we found that AX still decreased the mitochondrial ^∙^O_2_^−^ production levels in the 14-day-old animals of the *age-1* mutant but not the *daf-16* null mutant. There was no significant difference in the mitochondrial ^∙^O_2_^−^ levels after 14 days from hatching in the AX-treated wild-type N2 compared with the 4-day-old animals. The AX treatment did not decrease but increased the mitochondrial ^∙^O_2_^−^ production levels of the 4-day-old animals of N2 and *age-1* (Figure 3(a)). However, the AX-dependent increases in the mitochondrial ^∙^O_2_^−^ levels were not significant in the AX-treated 14-day-old animals of N2 and *age-1* (Figure 3(b)). In general, dose ranging revealed that the mitochondrial ^∙^O_2_^−^ production levels per mg of mitochondrial protein in the 14-day-old animals, at approximately 150–300 × 10^3^ the relative luminescence intensity unit (RLU) per second, was significantly enriched compared to the values in the 4-day-old animals (Figures 3(a) and 3(b)). 

**Figure 4:**
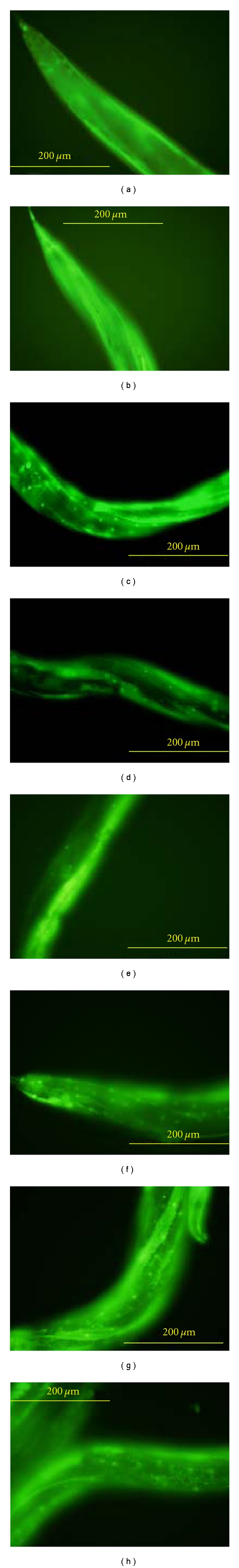
Localization of DAF-16::GFP in wild-type (a, b, e, and f) and *age-1* animals (c, d, g, and h). Panels of (a, b, c, and d) show the 4-day-old animals, and panels of (e, f, g, and h) show the 14-day-old animals. Furthermore, Panels of (b, d, f, and h) show AX-exposed animals in each strain. Scale bar = 200 *μ*m. Means of number of DAF-16-translocated nuclei ± SD in control and 1 mM AX were as Table 2.

In the 4-day-old animals of wild-type N2, almost all DAF-16 protein was observed in the cytoplasm and not the nucleus. This phenomenon was also similar in the AX-exposed 4-day-old animals (Figures 4(a) and 4(b)). In contrast, the DAF-16 translocation into the nucleus had already been observed in the 4-day-old animals of *age-1* with and without AX. The DAF-16-translocated nuclei were mainly in the epithelia, musculature, intestine, and part of the nervous system (Figures 4(c) and 4(d)). On the other hand, the DAF-16 was more localized in the nuclei of the 14-day-old animals of N2, and the translocation was significantly enhanced in the AX-exposed animals (Figures 4(e), 4(f), and Table 2). In the 14-day-old animals of *age-1*, more DAF-16 was translocated into the cytoplasm from the nucleus of the musculature and intestine compared to the 4-day-old animals (Figures 4(c), 4(d), 4(g), and 4(h)).

## 3. Discussion

Based on exposure of AX to several strains, we observed a longevity effect of about 16–30% in the wild-type N2 and long-lived mutant *age-1* of *C. elegans*. In contrast, no significant differences in the AX-dependent lifespan extension were noted in a *daf-16* null mutant. AX exposure to wild-type and *age-1* animals mainly enhanced the mRNA expression of the DAF-16 target genes and increased the nuclear localization of the DAF-16 transcription factor. Furthermore, it was shown that AX also caused a decrease in the mitochondrial ROS production during the long-term exposure to these animals. This finding suggests that AX indirectly protects intracellular organelles, such as mitochondria and nuclei, from oxidative damage during normal aging because the AX molecules at the surface and inside the phospholipid membranes in intracellular organelles have dual activities to quench ^1^O_2_ and scavenge lipid peroxidation by free radicals [25]. That is, the mitochondria protected from oxidative damage leak less ROS during the mitochondrial respiration, and the nuclei protected from oxidative damage are more active for the gene expression in organisms. It is likely that the oxidative stress-induced expression of antioxidant genes has been continued at least until 14 days old in the AX-treated animals. Moreover, we propose that AX protects the intracellular organelles through the bioactivities at the phospholipid membranes of cells (including the mitochondrial and nuclear membranes) [25] and increases the expression of the DAF-16 target genes via the Ins/IGF-1 signaling pathway, at least in part, in the nematode *C. elegans*. As a result, AX increases the lifespans of the wild-type and long-lived *age-1* mutant, which has activated the Ins/IGF-1 signaling. In particular, attention is directed to a more effective AX-dependent lifespan extension in the wild-type rather than the Ins/IGF-1 signaling-activated strain. 

On the other hand, it is interesting that the functionality of the carotenoids (such as AX) is determined by its subcellular localization [29]. Studies using domestic animals have shown a significant uptake of orally fed carotenoids, for example, lutein and *β*-carotene, by the microsomes, cytosol, and nuclei of the circulating peripheral blood leukocytes with the mitochondria showing the highest uptake in the animals [30, 31]. In a recent paper, it was reported that AX had accumulated in the mitochondria of normal human mesangial cells cultured with a high concentration of glucose and reduced the production of the mitochondrial ROS-modified proteins [32]. The mitochondrial respiratory chain system utilizes approximately 85% of the oxygen consumed by the cell to generate ATP; therefore, an intracellular organelle mitochondrion is the most important source of ROS [33]. Thus, the mitochondria are a key player for lifespan determination in organisms based on the mitochondrial oxidative stress theory of aging [1, 2, 34]. Accordingly, the localization of the carotenoids in the mitochondria has been of particular relevance based on the previous reports. AX prevented the lipid hydroperoxide (LOOH) generation in membrane liposomes enriched with polyunsaturated fatty acids and improved the muscle lipid metabolism under the ROS generation in exercise groups of mice [35, 36]. Notably, McNulty et al. inferred that AX preserved the membrane structure and exhibited a significant antioxidant activity because AX showed a significant reduction in lipid peroxidation rather than other apolar carotenoids, such as lycopene and *β*-carotene [35]. In addition to this dual antioxidant capacity (quenching of ^1^O_2_ and scavenging of lipid peroxidation), the direct ^∙^O_2_^−^ scavenging efficiency of AX delivered in the DMSO vehicle was evaluated using an *in vitro* isolated human neutrophil assay [29]. Likewise, our study indicated that supplemental DMSO-dissolved AX delivered into the nematode plays a role regarding some antioxidant properties in the mitochondrial and nuclear membranes without modification of the constituent lipid structure under stressful conditions during normal aging. Of course, it is expected that these physical properties of AX against cellular and intracellular membranes are effective even in the *daf-16* null mutant used in the current study. However, we consider that an imbalance during the production and quenching of ROS had occurred in the mitochondria of the AX-treated *daf-16* mutant because not only the antioxidant genes but also the mitochondrial metabolic genes were regulated as the targets of DAF-16 transcription factor and related to the *C. elegans* lifespan and metabolism [37]. 

 Recently, Miyashita has suggested that carotenoids have other novel biological activities, which are independent of the antioxidant properties. Modulation of the transcription activity of carotenoids is known to have an anticancer effect; however, the underlying mechanisms of this action still remain uncertain [26]. Moreover, the nonprovitamin A carotenoids (such as lutein, cantaxanthin, lycopen, and AX) are also capable of altering the patterns of gene and protein expressions and have a cellular function with a specific nutritional impact on the body [30, 38]. 

In summary, we conclude that AX taken into the subcellular organelles in nematode *C. elegans* consequently protects the cells at the surface and inside lipid-rich membranes against oxidative injury and functions to keep the optimal intracellular ROS balance mediated by regulation of the DAF-16 targets via the Ins/IGF-1 signaling pathway during normal aging. Hence, AX as a potential *in vivo* supplemental agent, extends the lifespan of nematodes not only by the direct antioxidant activities but also via the indirect AX-related activation of the Ins/IGF-1 signaling.

## 4. Methods

### 4.1. Materials

 The *C. elegans* strains, wild-type N2 var. Bristol, *age-1(hx546)*, and *daf-16(mgDf50)* were obtained from the Caenorhabditis Genetics Center at the University of Minnesota (Minneapolis, Minn, USA). The *age-1(hx546)* mutant is the first long-lived strain [39, 40], and the *daf-16(mgDf50)* mutant has a deficiency completely eliminating the *daf-16* coding region [41]. Worms were grown at 20°C on nematode growth medium (NGM) agar plates with *E. coli *[42–44]. 

### 4.2. Measurements of AX-Treated Lifespan

**Figure 5:**
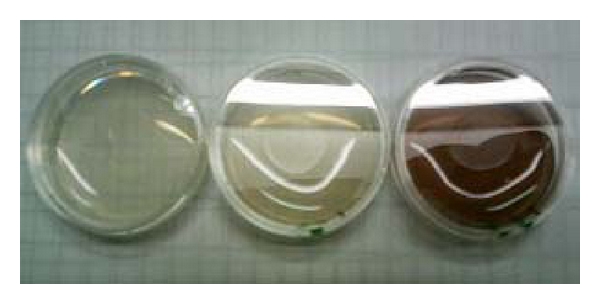
AX-containing NGM plates for measurement of lifespan in nematode. Left-, middle-, and right-hand plates contain a red carotenoid pigment AX of 0, 0.1, and 1 mM, respectively. For its lipid solubility, nonesterified AX crystalline was delivered using a DMSO vehicle in NGM [29].

 The gravid hermaphrodites from the NGM agar plates were washed then dissolved in alkaline sodium hypochlorite in order to collect the eggs in utero. The released eggs were allowed to hatch by overnight incubation at 20°C in S buffer to the age synchronous cultures of the L1 stage larvae [44]. The lifespan of the hermaphrodites at 20°C was measured with or without AX crystalline (Santa Cruz Biotechnology, Inc., Santa Cruz, Calif, USA) solubilized in dimethyl sulfoxide (DMSO; Sigma Chemical Co., St. Louis, Mo, USA) (Figure 5) [29]. In order to prevent progeny production, 5-fluoro-2′-deoxyuridine (FUdR; Wako Pure Chemical Industries Ltd., Osaka, Japan) was added to the NGM agar plate at the final concentration of 40 *μ*M after the animals had reached adulthood [43]. 

### 4.3. Quantitative RT-PCR for Antioxidant Enzymes

 The poly(A)^+^ RNA of the animals cultured with or without AX was prepared, and then the cDNA was synthesized using a reverse transcription reaction [42]. The cDNA was used as a template for the subsequent polymerase chain reactions (PCR). We carried out the PCR for five *sod* and two *ctl* genes in the 14-day-old animals. Fragments of the PCR products were confirmed by agarose gel electrophoresis and ethidium bromide (EtBr). The fluorescence intensity of EtBr in the DNA fragments was half-quantitatively measured using a LAS-4000UVmini luminescent image analyzer (Fujifilm Co., Tokyo, Japan). The expression data were normalized to each transcript level of the *act-1* gene (encoding the body wall and pharyngeal muscle actin protein) in N2, *age-1(hx546)*, and *daf-16(mgDf50)*. 

### 4.4. Measurements of Mitochondrial ^∙^O_2_^−^ Production

 For isolation of the mitochondria fraction, the 4- and 14-day-old animals were treated as previously described [28]. The mitochondria fraction was resuspended in the TE buffer. The protein content of each fraction was determined using a BCA Protein Assay Kit (Pierce Biotechnology, Inc., Rockford, Ill, USA). The mitochondrial ^∙^O_2_^−^ production was measured using the specific chemiluminescent probe, 2-methyl-6-p-methoxyphenylethynyl imidazopyrazinone (MPEC; ATTO Co., Tokyo, Japan) [45]. Forty *μ*g of the intact mitochondria in 1 mL of the assay buffer containing 0.7 *μ*M MPEC was placed in an AccuFLEX Lumi 400 luminometer (Aloka Co., Ltd., Tokyo, Japan), and the relative luminescence intensity per second was measured. 

### 4.5. Subcellular Localization of DAF-16

 To detect the intracellular DAF-16 activity, pGP30 vector (obtained from Dr. T. E. Johnson's laboratory), which has a construct fuged the *daf-16* gene transcript a2 (*daf-16*a2) to *gfp* gene, was microinjected into each gonad of the wild-type and *age-1* animals at 100 ng/*μ*L with pRF4 containing the *rol-6(su1006)* gene. The presence or absence of DAF-16 localization into the nucleus of the 1 mM AX-exposed transgenic 4- and 14-day-old animals was observed using an Olympus Fluorescence Microscope with Digital Imaging System BX51TRF (Olympus Co., Tokyo, Japan).

## Figures and Tables

**Table 1:** Effect of AX on mean and maximum lifespans at 20°C in several age-related mutants.

Strain (condition)	Mean lifespan (days)			Max. lifespan (days)
N2 (control)	31.5 ± 6.4	25.5 ± 6.0	24.5 ± 5.1	43.8 ± 4.8
N2 (0.1 mM AX)	38.4 ± 5.3**	32.2 ± 9.1**	27.7 ± 5.7**	51.6 ± 6.0*
N2 (1 mM AX)	41.4 ± 6.5**	32.8 ± 8.7**	28.6 ± 6.4**	53.3 ± 6.1*
*age-1 *(control)	47.1 ± 15.1	40.1 ± 14.6	46.4 ± 14.7	77.6 ± 7.0
*age-1* (0.1 mM AX)	50.2 ± 16.1	50.2 ± 16.8**	50.5 ± 13.1*	80.0 ± 7.8
*age-1 *(1 mM AX)	54.8 ± 14.7**	49.8 ± 17.6**	51.4 ± 12.9*	82.6 ± 8.2
*daf-16 *(control)	19.2 ± 2.8	19.3 ± 2.9	18.0 ± 4.2	24.6 ± 2.6
*daf-16* (0.1 mM AX)	21.2 ± 4.0	20.1 ± 2.8	18.6 ± 3.5	27.6 ± 3.5
*daf-16* (1 mM AX)	22.0 ± 4.7	19.6 ± 3.5	19.5 ± 2.9	28.6 ± 3.8

Results about mean lifespan are indicated as means ± SD from three independent experiments. Results about maximum lifespan are expressed as means ± SD of more than six determinations. *P* values by *t*-test with an asterisk (controls versus AX-treated conditions) significantly differ as follows; *P < 0.05 and **P < 0.001.

**Table 2:** Effect of AX on DAF-16 localization into the nucleus in several age-related mutants.

Strain (condition)	Age (days)	Number of DAF-16-translocated nuclei (/unit area)
N2 (control)	4	N.D.
N2 (1 mM AX)	4	N.D.
N2 (control)	14	3.4 ± 0.5
N2 (1 mM AX)	14	12.0 ± 2.0*
*age-1 *(control)	4	12.6 ± 1.1
*age-1* (1 mM AX)	4	13.6 ± 3.2
*age-1 *(control)	14	14.2 ± 2.3
*age-1* (1 mM AX)	14	13.6 ± 1.3

DAF-16-translocated nuclei were counted mainly in the epithelia, musculature, and intestine. N.D. indicated not detected. Results about the number of DAF-16-translocated nuclei are indicated as means ± SD from more than five independent transgenic animals. *P* values by *t*-test with an asterisk (controls versus AX-treated condition) significantly differ as follow; *P < 0.001.
